# Comparative Evaluation of the Efficacy of TheraCal LC, Mineral Trioxide Aggregate, and Biodentine As Direct Pulp Capping Materials in Patients With Pulpal Exposure in Posterior Teeth: A Triple-Blinded Randomized Parallel Group Clinical Trial

**DOI:** 10.7759/cureus.55022

**Published:** 2024-02-27

**Authors:** Joyeeta Mahapatra, Pradnya P Nikhade, Aditya Patel, Nikhil Mankar, Prachi Taori

**Affiliations:** 1 Department of Conservative Dentistry and Endodontics, Sharad Pawar Dental College and Hospital, Datta Meghe Institute of Higher Education and Research, Wardha, IND

**Keywords:** calcified barrier, direct pulp capping, biodentine, mineral trioxide aggregate, theracal lc

## Abstract

Background

The aim was to evaluate and compare the efficacy of TheraCal LC, mineral trioxide aggregate (MTA), and Biodentine as direct pulp capping (DPC) materials in patients with pulpal exposure in the posterior teeth.

Methodology

A total of 54 samples were assessed for eligibility. Out of this, 12 teeth samples failed to meet the inclusion criteria. Finally, 42 teeth samples were selected which were randomly distributed into three groups (n = 14). Groups A, B, and C received the intervention of MTA, Biodentine, and TheraCal LC, respectively. The assessment was performed clinically to check for postoperative pain, tenderness, and neural sensibility, and the radiographs were used to check the presence of periodontal ligament (PDL) space widening, calcified barrier, and periapical radiolucency at the follow-up of 21 days, three months, and 12 months. The outcomes depended on the clinical and radiographic success rates recorded at 12 months of recall.

Results

Overall successful outcome of DPC clinically at different periods was 97.61% at three months and 88.09% at 12 months. A Chi-square test was used which showed that the difference was statistically nonsignificant. For groups A, B, and C, the success rate at follow-up came out to be 85.71%, 100%, and 78.57% at 12 months, respectively.

The overall radiographic success rate of DPC at different time periods was 83.33% at three months and 88.09% at 12 months. A Chi-square test was used which showed that the difference was statistically nonsignificant. For groups A, B, and C, the success rate at follow-up came out to be 85.71%, 100%, and 78.57% at 12 months, respectively.

Conclusion

Resin-based calcium-silicate agent (TheraCal LC) showed good efficacy and can be used in practice with the predictability of a good success rate both clinically and radiographically. Thus, TheraCal LC can be utilized as an alternative to MTA or Biodentine in clinical practice, with the predictability of similar successful outcomes in patients with pulpal exposure in the posterior teeth.

## Introduction

The oral cavity is the second major diversified hub of microorganisms in the human body, constituting over seven hundred species of bacteria [1]. Exposure of the pulpal chamber of a tooth to this oral environment leads to a microbial invasion of the pulp-dentin complex, resulting in their breakdown [2]. The various etiologies responsible for such pulp exposures are traumatic impact, caries, or mechanical exposures due to iatrogenic errors [3]. If such exposures of the pulp are not timely treated, it can progress to apical periodontitis followed by other periapical lesions that would require invasive procedures like root canal treatment or even extraction.

The primary objective of vital pulp therapy is the preservation of the tooth pulp by removing any tissue contamination caused by the ingression of bacteria and the promotion of repair/replacement of the calcified tissue barrier that is histologically identified as the dentin bridge [4]. Direct pulp capping (DPC) is one technique where a pulp protective agent is applied at the exposed area, like a protective wound dressing to maintain a vital pulp and restore its healthy state [5-7]. It has a varying degree of success and failure rates which are influenced by the diverse procedural approaches and selection of the advanced materials available to a clinician [8].

Calcium silicate materials (CSMs) are the new upcoming materials that are still being experimented for their extensive use in dentistry. It has various favorable applications in this field such as root-end filling, repairing perforated tooth structure, as a sealant for obturation, in pulp capping, and pulpotomy. These cements exhibit certain desirable characteristics like adequate sealing capacity by chemically bonding to the tooth structure, antimicrobial activity, biocompatibility, bioactivity with the ability to stimulate and modulate tissue formation, resistance to moisture sensitivity, nonabsorbability to tissue fluid, dimensional stability, good strength, radio-opacity, and easy manipulation [9].

Mineral trioxide aggregate (MTA) is the first generation of CSMs which is known to have a good clinical success in cases that required repairing of perforation in the furcation area and for retrograde restorations. It shows good compatibility to the peri-radicular and periodontal tissues with evidence of minimal inflammation [10]. Biodentine comprises an enhanced calcium silicate-based material. It has excellent biocompatibility, improved mechanical properties, and excellent bioactive behavior and does not discolor the tooth [11]. TheraCal LC is a CSM present in syringes in pasty consistency. It can be used as a DPC material as well as a protective liner [12].

The current study evaluated and compared the efficacy of MTA, Biodentine, and TheraCal LC as DPC agents in patients with pulp exposure in the posterior teeth. The objective of the study was to evaluate the efficacy of each of the pulp capping agent, i.e., MTA, Biodentine, and TheraCal LC, as DPC agent using the clinical parameters of postoperative pain, tenderness on percussion, neural sensibility, and radiographic parameters of the presence of calcified barrier, periodontal ligament (PDL) space widening, and periapical radiolucency at recall period of 21 days, three months, and 12 months. The second objective had been the comparison of efficacy of MTA, Biodentine, and TheraCal LC as DPC agents with each other in relation to the abovementioned parameters.

## Materials and methods

Sample size calculation

Based on previous studies, and considering the success rate of DPC using MTA as 80% [7] versus calcium hydroxide as 33.3% [13] using α = 5% with the power of 80%, the sample size was calculated with the formula n = [Zα/2 +Z1-β]2 [p1 (1-p1) + p2(1-p2)]/(p1-p2) 2. Thus, the sample size was 14 for each group, i.e., a total of 42 samples that were needed.

Study design

A randomized, parallel-group clinical trial was conducted in the Department of Conservative Dentistry and Endodontics, Sharad Pawar Dental College and Hospital, Wardha. The study got its approval from the Institutional Ethics Committee of Datta Meghe Institute of Medical Sciences (Deemed to be University) [Ref No.- DMIMS(DU)/IEC/Aug-2019/8279] and was registered at the Clinical Trial Registry-India as CTRI/2020/01/023047.

The recruitment of patients was done from the population that reported to the outpatient department (OPD). Each of them had been educated about the importance of DPC which included the potential risks and benefits of the intervention before they were enrolled in the clinical trial. The potential risks include the development of postoperative pain and flare-ups. Written informed consent was obtained before the start of the intervention. Their demographic details were confidential. The generation of the sequence for using the different agents was computer-generated randomly using MS Excel (Microsoft Corporation, Redmond, Washington, United States).

The samples were distributed to three different treatment groups. In group A, MTA was used. In group B, Biodentine was used. In group C, TheraCal LC was used.

**Figure 1:**
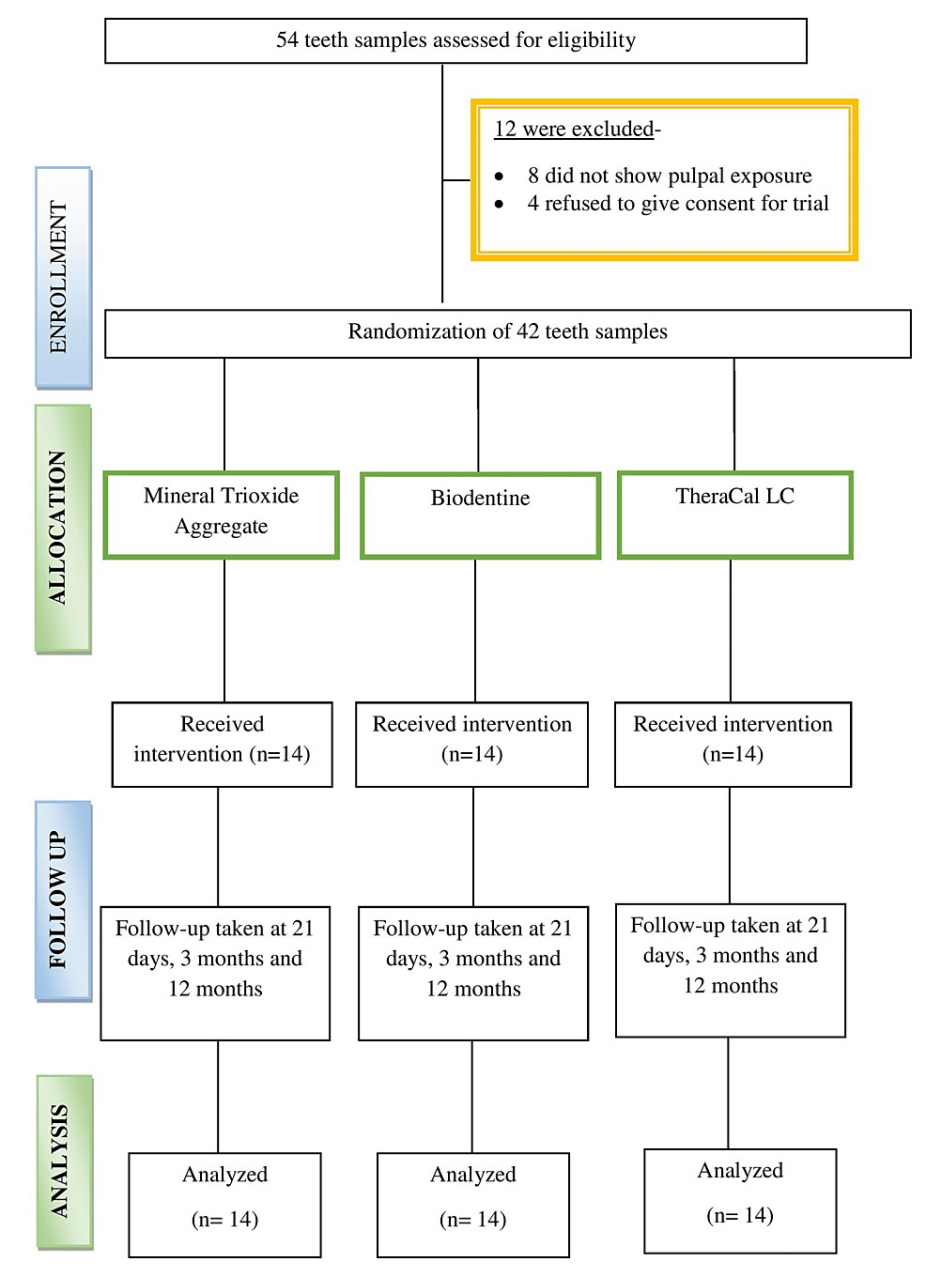
The Consolidated Standards of Reporting Trial (CONSORT) flowchart of participants throughout the trial

The allocation of the materials was centralized. There was a third party who received the random allocation list and revealed the operative personnel regarding the sequence of the DPC material just before the placement of the material. Patient recruitment was done by a different investigator. The point to be noted here was that the appearance, handling techniques, and manipulation of different DPC agents used in this research were very different from each other. Therefore, operator blinding was not feasible for this study. However, participant blinding was carried out. So, the patients were unaware of their allotment to the different treatment groups. The operator who undertook the treatment procedure did not take part in assessing the outcome. The outcome assessment was conducted by a blinded investigator who evaluated the patient both clinically as well as radiographically. Statistician blinding was also done. So, this was a triple blinded study. The trial has been reported as per the Consolidated Standards of Reporting Trials (CONSORT) 2010 statement: updated guidelines for reporting parallel group randomized trials (Figure 1).

Inclusion Criteria

Patients aged 17-40 years were included in the study. Only mature permanent teeth with carious extension in dentin in more than half or three-fourth the thickness of the dentin bulk, as observed on an intraoral periapical radiograph (IOPA) were included [14]. Also, those teeth that gave a positive response similar to that of the contralateral control tooth to cold test (using Refrigerant spray: Endo Frost), which subsides within 1-2 s after removal of the stimulus, thus confirming the diagnosis of reversible pulpitis, were included.

Exclusion Criteria

This study excluded primary teeth, tooth not showing any pulp exposure following complete caries removal, tooth with pulpal necrosis, tooth diagnosed as irreversible pulpitis, tooth with periapical lesion as observed radiographically, tooth with periodontitis, tooth with cracks, tooth displaying internal or external resorption, tooth with calcified canals, tooth in which pulpal hemostasis was unachievable even after the accidental pinpoint exposure, patients with systemic disease or those who are immunocompromised, pregnant patients, and patients on antibiotics or analgesics course within one week before the start of the trial.

Clinical procedure

A detailed case history had been recorded, and a thorough evaluation was done clinically and radiographically. The experience of pain by the patients was recorded with the help of Visual Analogue Scale (VAS). Local anesthetic solution (lignocaine 2% with epinephrine 1:200,000 solution) was injected for the concerned tooth followed by its rubber dam isolation [15]. With the help of an air-rotor handpiece under slow speed, using round bur, the superficial carious layer had been excavated. Cavity had then been disinfected using 2% chlorhexidine, following which there was thorough irrigation using 0.9% saline. The remaining soft caries in the deeper layers had been excavated carefully with spoon excavator. Cavity disinfection was done by irrigating it with 3% sodium hypochlorite (NaOCl). Excess NaOCl was rinsed off from the cavity using saline. The rest of the NaOCl is soak-dried from the cavity with cotton pellets [14]. The pulp capping agent was selected and was used in patient according to their allotment in the different groups as per the randomization table.

In patients allotted to group A, MTA (ProRooT MTA, Dentsply, Germany) was used. It was available in powder and liquid form. They were dispensed in a mixing pad in the proportion of liquid/powder ratio of 1:3, i.e., 0.33 g water to 1 g powder [3]. The material mass was then placed at pulp exposure site with the help of a sterile carrier. Patient had been recalled after 24 hours for the final restorative procedure.

In group B, Biodentine (Septodont Biodentine, France) was used. Here, the powder and the liquid are dispensed in a mixing paper pad in proportion as instructed by the manufacturer (for one capsule of Biodentine containing 700 mg of powder, 5 drops of Biodentine liquid is recommended) [16]. The mix was placed on the exposed site using a carrier and then final restoration was undertaken on the same day.

In group C, TheraCal LC (Bisco Inc., Schaumburg, IL, USA) was used. The material was available in a syringe in a paste form. It was directly dispensed on the affected site after achieving a fairly dry surface of at least 1 mm or less in width and 1.7 mm in thickness [17]. It is then light cured for 20 s [18]. This was followed by the final restoration.

In the final restorative procedure, a base of glass ionomer cement (GIC) (GC Fuji, Japan) was given followed by a composite restoration (Spectrum Microhybrid composite, Dentsply, USA). Following completion of the treatment, an IOPA of the concerned tooth had been recorded and set at 70 kVp, 8 mA with exposure time of 0.4 s. The IOPA had been kept as a baseline for the future recall radiographs [14].

Recall and outcome measures

Clinical examination of the concerned tooth included the presence/absence of postoperative pain, tenderness on percussion, and neural sensibility to cold test, whereas and radiographical examination of the tooth included checking for the presence/absence of PDL space widening, calcified barrier at the restored site, and periapical radiolucency as observed on the IOPA. These examinations were performed at the recall interval of 21 days, three months, and 12 months.

The primary success rate was determined by the presence of a calcific barrier adjacent to the capped area in the pulpal direction as observed on the IOPA. The secondary success rate had been determined by the absence of postoperative pain, tenderness on percussion, positive response to neural sensibility test (cold test), and the absence of any radiographical signs of PDL space widening and the absence of any periapical radiolucency.

The pain of the patients was recorded with the help of VAS [14]. Assessing the tenderness had been conducted by vertical percussion of the restored tooth. Refrigerant spray (Endo Frost™, Roeko, Langenau, Germany) was used for performing cold test to check neural sensibility.

The presence of PDL space widening was defined as "less than double that of the equivalent healthy PDL space of the adjacent healthy tooth" [19]. According to Low et al. (2008) and Bornstein et al. (2011), the presence of periapical radiolucency is affirmed when there is a radiolucent area present in association with the radiographic apex of the root of the concerned tooth ≥2 times the width of the PDL space [20,21].

Statistical analysis

The normality of data distribution had been analyzed using the Shapiro-Wilk test. Chi-square test had been applied for dichotomous variables. The one-way analysis of variance test had been utilized for the parameter of pain to check the mean differences between the groups at different time period. The software used was the IBM SPSS Statistics for Windows, Version 24.0 (Released 2016; IBM Corp., Armonk, New York, United States).

## Results

A total of 54 teeth samples had been considered for our study. Out of these, eight did not show any exposure of the pulpal chamber after caries excavation, and four of the patients denied giving consent for participation. A total sample size of 42 teeth was, thus, eventually recruited for the trial. They were randomly allocated to three groups (n = 14), i.e., group A, group B, and group C. Regarding the gender characteristic, the difference was statistically nonsignificant between the groups (p-value = 0.47) (Table 1).

**Table 1:** Demographic data of study recruits

Groups	Sex	
	Males	Females
A (n = 14)	3	11
B (n = 14)	6	8
C (n = 14)	5	9
p-value	0.47	

In terms of age, the mean and standard deviation for groups A, B, and C are 27.28 ± 6.15 years, 27 ± 7.47 years, and 26.85 ± 5.81 years, respectively. In terms of the type of teeth, out of the 42 teeth, seven were premolars (16.6%), 23 were first molars (54.8%), and 12 were second molars (28.6%).

The various periods during which the evaluation of different clinical and radiographic parameters was performed are denoted as T-1, recall immediately before the procedure; T0, recall immediately after the procedure (baseline); T1, recall after 21 days; T2, recall after three months; and T3, recall after 12 months.

Based on the formation of the calcified barrier at the site of the DPC, groups A, B, and C had shown a success rate of 85.71%, 100%, and 78.57% at the end of one year, respectively. Based on the presence of the PDL space widening of the concerned teeth with different pulp capping agent, groups A and C had shown PDL space widening by 14.28% and 21.42% at the end of one year, respectively. Group B had shown no PDL space widening at all the time periods of recall. Based on the presence of the periapical radiolucency of the concerned teeth, groups A and C had shown periapical radiolucency by 7.14% and 14.28%, respectively, at the end of one year. Group B had shown no periapical radiolucency at any of the time periods of recall.

Table 2 depicts the pain values of group A at various time intervals of recall, i.e., at three months and 12 months. The scores were 0.14 ± 0.53 and 0.57 ± 1.45, respectively. The difference was not statistically insignificant (p-value = 0.22).

**Table 2:** The mean and standard deviation value of pain score in group A at different recall periods and the comparison of these scores among them at different time intervals of recall T1, recall after 21 days; T2, recall after three months; T3, recall after 12 months; group A: MTA

Group	T1	T2	T3
A	0	0.14 ± 0.53	0.57 ± 1.45
F value	1.55		
p-value	0.22`		

Table 3 depicts the pain values of group B at various time intervals of recall, i.e., at 21 days, three months, and 12 months. 

**Table 3:** The mean and standard deviation value of pain score in group B at different recall periods and the comparison of these scores among them at different time intervals of recall T1, Rrecall after 21 days; T2, recall after three months; T3, recall after 12 months; group B: Biodentine

Group	T1	T2	T3
B	0	0	0
F value	0		
p-value	1		

Table 4 depicts the pain values of group C at various time intervals of recall. The score at 12 months was 0.71 ± 1.48. The difference was not statistically significant (p-value = 0.06). 

**Table 4:** The mean and standard deviation value of pain score in group C at different recall periods and the comparison of these scores among them at different time intervals of recall T1, recall after 21 days; T2, recall after three months; T3, recall after 12 months; group C: TheraCal LC

Group	T1	T2	T3
C	0	0	0.71 ± 1.48
F value	3.21		
p-value	0.06		

Based on the presence of tenderness on percussion of the concerned teeth with different pulp capping agent, groups A and C had shown tenderness on percussion by 14.28% and 21.42% at the end of 12 months, respectively. Group B had shown no periapical radiolucency at any of the time periods of recall. Based on the presence of the response to neural sensibility test of the concerned teeth with different pulp capping agent, groups A and C had shown response by 85.71% and 78.57%, respectively. Group B had shown response to neural sensibility test by 100% at all recall period interval.

Overall clinical and radiographic success rate

Table 5 depicts the overall clinical success rate of the DPC at different time periods which was 88.09% at 12 months.

**Table 5:** Intergroup comparison for the overall clinical success rate at different time periods T1, recall after 21 days; T2, recall after three months; T3, recall after 12 months; group A: MTA; group B: Biodentine; group C: TheraCal LC

Group	T1	T2	T3
A	14 (100%)	13 (92.85%)	12 (85.71%)
B	14 (100%)	14(100%)	14 (100%)
C	14 (100%)	14 (100%)	11 (78.57%)
Total	42/42 (100%)	41/42 (97.61%)	37/42 (88.09%)
Chi-square value	0.27		
p-value	0.99 (Not Significant)		

Table 6 depicts the overall radiographic success rate of the DPC at different time periods which was 88.09% at 12 months.

**Table 6:** Intergroup comparison for the overall radiographic success rate T1, recall after 21 days; T2, recall after three months; T3, recall after 12 months; group A: MTA; group B: Biodentine; group C: TheraCal LC

Group	T1	T2	T3
A	0	11 (78.57%)	12 (85.71%)
B	0	13 (92.86%)	14 (100%)
C	0	11 (78.57%)	11 (78.57%)
Total	0/42 (0%)	35/42 (83.33%)	37/42 (88.09%)
Chi-Square Value	0.02		
p-value	0.99 (Not Significant)		

## Discussion

DPC comprises the use of an agent with a good property of biocompatibility at the area of pulp exposure. This helps in inducing the production of a "hard tissue barrier" at the affected site which helps in preserving the vitality of the pulpal complex [22].

Baume and Holz (1981) conducted a clinical trial that concluded that an infected pulp has less chance of a successful outcome to treatment [23]. According to Ghoddusi et al. (2014), pulp capping done on carious pulp exposure showed a success rate of 87.5%-95.4%, whereas those restored after iatrogenic exposure had a success rate of 70%-98% [8].

According to Matsuo et al.(1996), patients under 40 years had better successful outcomes (85.7%) following DPC than those above 40 years of age (75%) in a 24-month recall [24]. Also, Marques et al. (2015) stated in their study that participants below 40 years have significantly higher successful outcomes than those above this age limit in a 12-month follow-up. The lower age limit is restricted to 17 years to make sure that only those tooth samples are included in the study which has closed apex. The posterior teeth up to the second molars undergo complete root formation by this age [25]. Young permanent teeth having open apices are excluded from the study because they have enhanced vascularity and healing as compared to mature permanent teeth. This will act as a confounding factor for inflammation and healing because vital pulp therapy relies on the host's immune response for dentin barrier formation.

Since there was a similarity in the rest of the clinical variables in all three groups, the type of DPC agent used was the only significantly affected factor in our research. In this clinical trial, the overall success rate of DPC came out to be 88.09% at the recall period of one year. As per the study of Awawdeh et al.(2018), the success rate of DPC at 12-month recall for MTA and Biodentine had been 100% and 96%, respectively, whereas at the end of 36 months, it was 96% and 91.7%, respectively [26]. Another study by Katge and Patil (2017) stated that the successful outcome of DPC at 12-month follow-up was 100% for both MTA and Biodentine. These findings are in accordance with the present trial where the difference was statistically insignificant between the groups at 12 months of recall [27].

The primary outcome measure of the current research had been the presence of the calcified barrier following DPC as observed radiographically. Toward the end of the 12-month follow-up, the presence of this barrier was 85.71% in the MTA group, 100% in the Biodentine group, and 78.57% in the TheraCal group. According to the study by Katge and Patil (2017), toward the end of the 12th month following DPC, an evident calcified barrier was seen in both MTA and Biodentine groups by 95.24% and 85.71%, respectively. They carried out their observation in periapical IOPA and termed this barrier as the "dentin bridge" [27]. Menon et al. (2016) radiographically observed a statistically significant intensification in the thickness of dentin at the site of DPC at the end of three months and six months when the values were compared to the baseline values in MTA and TheraCal groups. There was a significant dentin thickening when the values of a six-month follow-up were compared to that of a three-month follow-up. However, their study showed an insignificant difference when intergroup comparison of these values was carried out at all the follow-up intervals (baseline, three months, six months). They based their observation on bitewing radiographs[28]**. **Bakhtiar et al. (2017) histologically assessed the production of dentinal barrier following partial pulpotomy using ProRoot MTA, Biodentine, and TheraCal. At the eighth week, this value was 56%, 100%, and 11%, respectively. This disparity in the percentage of bridge formation was due to the differential hydration of the materials during setting reaction. The formation of the dentin bridge is dependent on two factors: hydration of the materials during setting reaction and calcium ion release from the set material[29]. Although TheraCal has high Ca+2 ion releasing property as compared to MTA and Biodentine [17], it has poor hydration properties. MTA and Biodentine set by formation of Ca(OH)_2_ which stimulates the releasing of transforming growth factor-b1. This factor recruits pulpal stem cells and stimulates odontoblastic differentiation that ultimately lead to this dentin barrier formation [30,31]. Incomplete hydration of TheraCal is due to the existence of resin in the composition like 2-hydroxyethyl methacrylate (HEMA), bisphenol A-glycidyl methacrylate (BisGMA), triethylene glycol dimethacrylate (TEGDMA), and urethane dimethacrylate (UDMA) that stay unpolymerized after contacting the pulpal tissue. They cause cytotoxicity to the pulpal fibroblasts by leaching monomers which hinders complete formation of the dentin barrier [32]. They also inhibit the secretion of "dentin sialoproteins" and "osteonectin" even in their nontoxic concentration [33]. These proteins take part in the mineralization process. Inhibition in their secretion leads to reduced mineralization in TheraCal group as compared to MTA and Biodentine groups.

Other radiographic parameters which are secondary outcome measures include the presence of PDL space widening and periapical radiolucency. The outcome of PDL space widening at 12-month follow-up for MTA and TheraCal came out to be 14.28% and 21.42%, respectively. No widening was seen in the Biodentine group. The outcome of the presence of periapical radiolucency at 12-month follow-up for MTA and TheraCal came out to be 7.14% and 14.28%, respectively. No periapical radiolucency was seen in the Biodentine group. According to a study by Carti and Oznurhan (2016), the radiographical successful outcome at 12-month recall of MTA and Biodentine groups came out to be 80% and 60%, respectively, while being utilized as pulpotomy material in deciduous molars [34].

One of the clinical outcome parameters was postoperative pain. Intragroup comparison of the pain score at different recall intervals of 21 days, three months, and 12 months in either of the three groups (MTA, Biodentine, or TheraCal) showing a statistically insignificant difference between their recall periods. Intergroup comparison of the pain score of different groups at various recall intervals (21 days, three months, and 12 months), too displayed a statistically nonsignificant difference among the groups. The second clinical parameter was the presence of tenderness on percussion which at the 12-month recall was 14.28% in the MTA group and 21.42% in the TheraCal group. This was completely absent in the Biodentine group at all time periods of recall. The third clinical parameter of the presence of neural sensibility at a 12-month recall was seen in MTA, Biodentine, and TheraCal groups by 85.71%, 100%, and 78.5%, respectively. Carti and Oznurhan (2016) determined the clinical success rate of DPC with MTA and Biodentine based on the above three clinical parameters [34]. During their 12-month recall, the success rate was 96% in each of the two groups, i.e., MTA and Biodentine. Kusum et al. (2015) determined the successful outcome clinically of MTA and Biodentine based on postoperative pain and the presence of tenderness on percussion. The success outcome at nine-month recall came out to be 100% in both the groups [35].

Hence, from the above results, it can be inferred that every DPC agent in the present trial displayed a good success rate, with Biodentine displaying the maximum efficacy and TheraCal LC showing the least efficacy. Sequence of successful outcome from higher to lower was Biodentine, MTA followed by TheraCal LC. At 12-month recall, the difference in successful outcome was statistically nonsignificant. The new resin-based calcium-silicate agent (TheraCal LC) showed good efficacy and can be used in practice with a predictability of good success rate both clinically and radiographically. The full trial protocol can be accessed at the below mentioned link: http://www.ijpronline.com/ViewArticleDetail.aspx?ID=16696 [36].

There were some limitations of the study. Firstly, the operator blinding was not possible as there was a variation in physical characteristics, appearance, and handling properties of the agents that was used. Then, there was subjectivity of the VAS. The maximum recall period was 12 months which is very short for evaluating the success rate of DPC materials. There may have been a reduction in the success rate had there been a longer period of recall [37,38].

There is scope for further studies which should be done to evaluate the success of DPC with newer pulp capping agents. Future studies can use newer parameters like determining the thickness of the dentin bridge. Long-term recall studies of three years or more can be performed for deriving the successful outcome of the agents that had been used.

## Conclusions

The DPC agent in the present trial displayed a good success rate, with Biodentine displaying the maximum efficacy and TheraCal LC showing the least efficacy. The sequence of successful outcomes from higher to lower was Biodentine, MTA followed by TheraCal LC. However, at 12-month recall, the difference in success rate was statistically nonsignificant. Thus, the new resin-based calcium-silicate agent (TheraCal LC) showed good efficacy and can be used in practice with the predictability of good success rate both clinically and radiographically. Therefore, within the limitations of the research, and according to the observations, the conclusion was derived that TheraCal LC can be utilized as an alternative to MTA or Biodentine in clinical practice, with the predictability of similar successful outcomes in patients with pulpal exposure in the posterior teeth.
